# Dendritic cell vaccination plus low-dose doxorubicin for the treatment of spontaneous canine hemangiosarcoma

**DOI:** 10.1038/s41417-019-0080-3

**Published:** 2019-01-23

**Authors:** V. Konduri, M. M. Halpert, Y. C. Baig, R. Coronado, J. R. Rodgers, J. M. Levitt, B. Cerroni, S. Piscoya, N. Wilson, L. DiBernardi, Z. Omarbekov, L. Seelhoff, V. Ravi, L. Douglass, W. K. Decker

**Affiliations:** 1 Department of Pathology & Immunology, Baylor College of Medicine, Houston, TX 77030 USA; 2 Lester Smith Medical Research Institute, San Antonio, TX 78229 USA; 3 Dan L. Duncan Comprehensive Cancer Center, Baylor College of Medicine, Houston, TX 77030 USA; 4 Scott Department of Urology, Baylor College of Medicine, Houston, TX 77040 USA; 5 Gulf Coast Veterinary Specialists, Houston, TX 77027 USA; 6 Sunset Boulevard Animal Clinic, Houston, TX 77005 USA; 7 Department of Sarcoma Medical Oncology, University of Texas MD Anderson Cancer Center, Houston, TX 77030 USA; 8 Center for Cell and Gene Therapy, Baylor College of Medicine, Houston, TX 77030 USA

**Keywords:** Medical research, Biological techniques

## Abstract

Angiosarcoma is a deadly neoplasm of the vascular endothelium. Metastatic disease is often present at diagnosis, and 5-year survival is only 10–35%. Although there exist no immunocompetent mouse models of angiosarcoma with which to study immune-based approaches to therapy, angiosarcoma is a major killer of companion dogs, responsible for up to 2% of all canine deaths in some susceptible breeds or an estimated 120,000 per year in the US. The canine disease (HSA) often presents in the spleen as acute hemoabdomen secondary to splenic rupture. Even if life-saving splenectomy is performed, median overall survival (OS) is only 48 days, and 1-year survival is negligible. Here we report the analysis of a pilot phase I open-label trial of chemo-immunotherapy performed on consecutively presenting splenectomized canines with histologically verified HSA. Subjects received an abbreviated course of low-dose doxorubicin plus alpha interferon and an autologous dendritic cell-therapy reported to enhance durable CD8^+^ memory. Disease was monitored monthly by abdominal ultrasound, chest X-ray, and echocardiogram. Median OS in the per protocol population was 109 days including one of five animals that died cancer-free at 16 months after documented resolution of relapsed disease. These results indicate that therapeutic administration of chemo-immunotherapy is both feasible and safe, substantiating the rationale for additional veterinary and human clinical studies.

## Introduction

Angiosarcoma is a deadly malignant neoplasm of the vascular endothelium that comprises ~2% of all soft tissue sarcomas. About half of angiosarcomas arise cutaneously in the dermal epithelium, with half of those located on the head and neck. Angiosarcoma has been poorly studied and little is known about the underlying molecular etiology of the disease. Prior radiotherapy is known to be an independent risk factor for the development of angiosarcoma, particularly in patients receiving prior radiotherapy for the treatment of breast cancer [1–3]. In the absence of prior radiotherapy, most cutaneous angiosarcomas are thought to arise spontaneously and are most commonly observed on the head and neck of elderly Caucasian men. Angiosarcoma is often multi-focal and/or locally advanced at presentation, rendering complete resection difficult. Despite the use of high-dose wide-field radiation and a variety of different chemotherapeutic regimens, relapse and metastatic spread are common. Five-year overall survival ranges from 10 to 35%, with median survival reported as 7 months to 2.6 years. Outcomes for patients with metastatic disease are significantly worse with virtually no long-term survivors [1–5]. Given the poor response of cutaneous angiosarcoma to standard of care therapies, there exists a compelling medical need to develop novel treatment regimens that can improve survival outcomes.

Immunotherapy of angiosarcoma is difficult to model in mice given that existing murine sarcoma models involve xenotransplantation of primary human tumors into immunocompromised animals. Modern genome-editing techniques have allowed generation of certain sarcomas with well-characterized molecular aberrations such as the Ews-Fli1 translocation associated with Ewing’s sarcoma or the Pax3-Fkhr translocation associated with alveolar rhabdomyosarcoma. Osteosarcoma models can also be reliably generated; however, the modeling of sarcomas with complex karyotypes and variable, low-penetrance genetics remains a significant challenge [6]. A recent report of a mouse angiosarcoma cell line derived from a spontaneous tumor in a Notch1^−/−^ background still requires re-implantation in a NOD/scid immunosuppressed host to exhibit tumorigenicity [7]. In contrast, hemangiosarcoma (the terminology used in veterinary medicine—canine hemangiosarcoma (HSA)) spontaneously arises in domestic canines at a greater frequency than any other known species with marked overrepresentation in German Shepherd, Golden Retriever, and Labrador breeds. HSA accounts for up to 2% of all canine tumors and 1% of all deaths among canines over the age of 10. Splenic hemangiosarcoma comprises up to half of all canine HSA and exhibits a median overall survival (OS) as low as only 48 days if treated solely by splenectomy. Adjuvant doxorubicin may increase median OS to 150 days; however, even with adjuvant chemotherapy, 1-year OS remains exceedingly poor [8–12].

A variety of clinical investigations have suggested that certain sarcomas may be amenable to treatment by active, specific immunotherapy. Dillman et al. [13] reported that sarcoma patients who became DTH positive (*n* = 8) following administration of irradiated, autologous tumor cells exhibited an OS of 16.6 months, whereas patients who remained DTH negative (*n* = 8) exhibited an OS of only 8.2 months. Sarcomas have also been treated by means of experimental dendritic cell vaccination. Dendritic cells (DC) are the master regulators of the adaptive immune response, and much effort has been focused upon the development of these specialized cells for use in therapeutic vaccination protocols [14]. In the largest sarcoma DC vaccine trial reported to date, 52 patients with recurrent or metastatic Ewing’s or alveolar rhabdomyosarcoma underwent apheresis after which 30 received DC pulsed with peptides corresponding to tumor-specific translocation breakpoints. At mean follow-up of 7.8 years, 40% of vaccinated patients (12/30) remained alive and disease-free. Five-year OS was 43% among vaccinated patients versus 31% among all patients enrolled [15]. In canines, there exists only a single report in which vaccine immunotherapy was used as an adjuvant treatment for splenic hemangiosarcoma. Among 13 dogs that received the full vaccination course of adjuvanted allogeneic HSA lysate, mean OS was 182 days; however, inclusion in the per protocol analysis required that enrolled animals survive long enough to receive all eight vaccinations, a period of 154 days [16]. Therefore interpretation of this impressive increase in survival is problematic.

Recent interrogation of a novel basic regulatory mechanism has expanded the paradigm by which regulation of cellular immunity is understood. This work has demonstrated that the loading of DC with antigenically similar (homologous) MHC class I and II antigens mediates a cell-intrinsic upregulation of T_H_1 polarization. This polarization consists of differential cytokine secretion, upregulation of surface costimulatory molecules, and preferential induction of IFN-γ-secreting CD8^+^ cytolytic T cells with enhanced memory capacity. DC loaded in this manner also demonstrate a substantial reorganization of the transcriptome, exhibiting a transcriptional profile significantly different from alternatively loaded DC in areas such as interferon signaling, antigen-presentation, innate antiviral response, protein ubiquitination, and DC licensing [17–24]. This concept has subsequently been utilized for the generation of cell-based vaccine platforms that specifically target cancer with highly cytotoxic T-cell responses of durable memory potential [22, 24]. Building upon previous work in which murine pancreatic ductal adenocarcinoma was treated by means of a similar approach [22], we adapted this dendritic cell platform for the treatment of canine splenic hemangiosarcoma. The aim of the study was to evaluate the feasibility and safety of chemo-immunotherapy while simultaneously validating the feasibility of spontaneous veterinary cancers in the dog for use as a model therapeutic system [25].

## Materials and methods

### Study animals and ethics statement

All companion canines enrolled in the study were bona fide clinical subjects that presented in study-affiliated veterinary clinics with histopathologically verifiable hemangiosarcoma. Study protocol AN-7705 was approved by and carried out in accordance with the recommendations of the Baylor College of Medicine Investigational Animal Care and Use Committee (IACUC). All owners of enrolled study animals were required to provide written informed consent and participate in a formal informed consent process during which all risks and benefits of study participation were explained in person. All study-related procedures were provided free of charge.

### Clinical protocol

#### Splenectomy

Spleen with associated tumor tissue was resected, and a section sufficient for definitive diagnosis was sent for histopathological confirmation of hemangiosarcoma. The remaining tissue was placed in a 50 ml conical tube with no additional liquid, flash frozen in liquid nitrogen, and stored at −80 °C until histopathology results could be confirmed.

#### Mobilization and blood collection

Upon determination of positive HSA histopathology, study animals were mobilized with rhG-CSF (Neupogen, Amgen, Thousand Oaks, CA). Five μg/kg Neupogen was administered subcutaneously every 12 h for 4 days (eight doses total) with a ninth dose of 10 μg/kg administered 12 h before collection. In total, 200 ml whole blood was drawn in a 250 ml heparinized collection bag (Terumo Medical Corporation, Somerset, NJ). Mobilized blood products were transported from the clinical site to the manufacturing site at ambient temperature in a temperature-controlled container. Administration of the vaccine was performed 7 days after collection.

#### Study day 0

Upon arrival at the veterinary clinic, DC vaccine aliquot one of four, suspended in 2 ml normal saline, was injected intraperitoneally under ultrasound guidance into the abdominal cavity in the vicinity of the original tumor. Concurrent with vaccine administration, five million units human interferon alpha (Intron A, Merck & Company, Inc, Kenilworth, NJ) was administered subcutaneously. Subsequently, five million units of veterinary type I interferon omega (Vibragen Omega, VioVet, Ltd, Bedfordshire, United Kingdom) was administered every other day for a total of 19 additional administrations. Echo/ultrasound of the heart was performed as were chest radiographs, complete blood count (CBC), and clinical chemistry.

#### Study day 3

A 2/3 standard of care dose of doxorubicin (20 mg/m^2^) was administered by slow i.v. infusion over the course of 2 hours.

#### Study day 10

DC vaccine aliquot two of four was thawed, washed, resuspended in two ml normal saline, and injected intraperitoneally into the abdominal cavity in the vicinity of the original tumor. Animals were observed for 2 hours following vaccine administration for signs of immune shock and/or cytokine storm.

#### Study day 17

A second 2/3 standard of care dose of doxorubicin (20 mg/m^2^) was administered by slow i.v. infusion over the course of 2 hours.

#### Study day 20

DC vaccine aliquot three of four was thawed, washed, resuspended in two ml normal saline, and injected intraperitoneally into the abdominal cavity in the vicinity of the original tumor. Animals were observed for 2 hours following vaccine administration for signs of immune shock and/or cytokine storm.

#### Study day 30

DC vaccine aliquot four of four was thawed, washed, resuspended in 2 ml normal saline and injected intraperitoneally into the abdominal cavity in the vicinity of the original tumor. Animals were observed for 2 hours following vaccine administration for signs of immune shock and/or cytokine storm. One animal with aggressive disease and an extra vaccine aliquot was administered a fifth vaccine dose on study day 40.

#### Additional analyses

CBC and abdominal ultrasound analysis were performed monthly between months 2 and 6 as well as on months 9 and 12 provided that the animals were still alive.

#### Post-mortem

Partial (i.e., lung and liver) or complete necropsy was performed if non-neoplastic death was suspected to validate the absence of malignancy.

### Canine DC vaccine generation

Mobilized peripheral blood was diluted with an equal volume of phosphate-buffered saline (PBS), and white cells were isolated by Ficoll (Lympholyte, Cedarlane Labs, Burlington, NC) density gradient centrifugation for 25 min at 450 × g. Following centrifugation, the upper layers of 50% autologous serum were collected and heat inactivated at 55 °C for 30 min. The middle layers comprising the white cell product were pooled, washed with PBS, and centrifuged at 600 × g for 10 min. Monocytes were collected by plastic adherence and cultured in AIM-V medium (ThermoFisher Scientific, Waltham, MA) supplemented with 10% autologous serum, 25 ng/ml rcGM-CSF (R&D Systems, Minneapolis, MN), 10 ng/ml canine rcIL-4 (R&D Systems), and 1% anti-anti (ThermoFisher) and incubated at 37 °C in a humidified chamber at 5% atmospheric CO_2_. Differentiating cells were supplemented with fresh media and cytokines on days 3 and 5. On day 6, immature DC were harvested as described previously [19] and loaded with tumor mRNA and lysate derived from the primary cryopreserved tumor tissue, also as described previously [19]. In brief, immature DC were resuspended at a concentration of 2 × 10^7^ cells/ml in Viaspan (Barr Laboratories, Pomona, NY) and incubated with tumor derived mRNA at a concentration of 1 μg mRNA/million DC for 10 min on ice in an electroporation cuvette with a 0.4 cm gap (Biorad, Hercules, CA). Cells were then electroporated at 250 V, 125 μF, and *Ω* = *∞* using a GenePulser Xcell (Biorad). Electroporated cells were then replated and incubated for 3 hours in the absence of cytokines in 2 mg/ml freeze–thawed tumor lysate. After incubation, cells were thoroughly washed with PBS and resuspended in AIM-V medium supplemented with 10% autologous serum, 25 ng/ml rcGM-CSF (R&D Systems), 10 ng/ml rcIL-4 (R&D Systems), 1% antibiotic–antimycotic (ThermoFisher), and a maturation cocktail consisting of 15 ng/ml rcIL-6 (R&D Systems), 10 ng/ml rcTNF-α (R&D Systems), 10 ng/ml rcIL-1β (R&D Systems), and 1 μg/ml PGE_2_ (Sigma-Aldrich, St. Louis, MO). After 24 h of maturation, cells were harvested, counted, and separated into four individual fractions. One fraction was resuspended in sterile PBS plus 10% autologous serum for injection, and the other three were cryopreserved. Approximately 40 million total vaccine cells per animal were administered in four separate vaccinations of approximately 10 million cells each, though numbers varied somewhat between animals.

### In vitro dendritic cell characterization

Canine DC were loaded with mRNA derived from an autologous HSA tumor in combination with either homologous tumor lysate or heterologous lysate derived from the murine RAW 264.7 myeloid cell line. DC were characterized by staining with anti-CD11c (eBioscience subsidiary of ThermoFisher), anti-CD40 (AbD Serotec subsidiary of BioRad Laboratories, Hercules, CA) and anti-CD80 (BioLegend, San Diego, CA) in conjunction with analysis by flow cytometry.

### Statistical analysis

Differences in survival between animals treated with surgery alone (historical control) and surgery plus chemo-immunotherapy were determined by Kaplan–Meier survival analysis using a large historical data set [11] as a control. To determine any degree of statistical significance, two sets of historical controls were used: surgery alone of animals with stage II and III HSA (two studies) [11, 12]; and surgery plus chemotherapy (three studies) [11, 16, 26]. By this methodology, the hazard rate from each controlled experiment was treated as a fixed value, but the observed hazard rate in any one simulation varied because the survival curve followed a random Poisson process. Each historical control was analyzed separately however, because comparisons all used the same experimental data and therefore were not considered to be independent. We first obtained a best estimate of the half-time of each study and treated this as coming from a single exponential decay such that lambda = ln0.5/half-time. In the case of the Wendelberg study [11] the weighted harmonic mean of the half-times of the stage II and III surgery-only animals was used to estimate the half-time of the combined group. We next used Monte–Carlo methods to generate a family of survival curves and count the number of curves from this family with half-times less than the experimental value of 109 days. We divided this value by the number of simulations to estimate the (one-sided) *p* value. Likewise, an empirical count of the 95% confidence range of the improvement was obtained from the family of survival curves.

## Results

### DC characterization in vitro

**Fig. 1:**
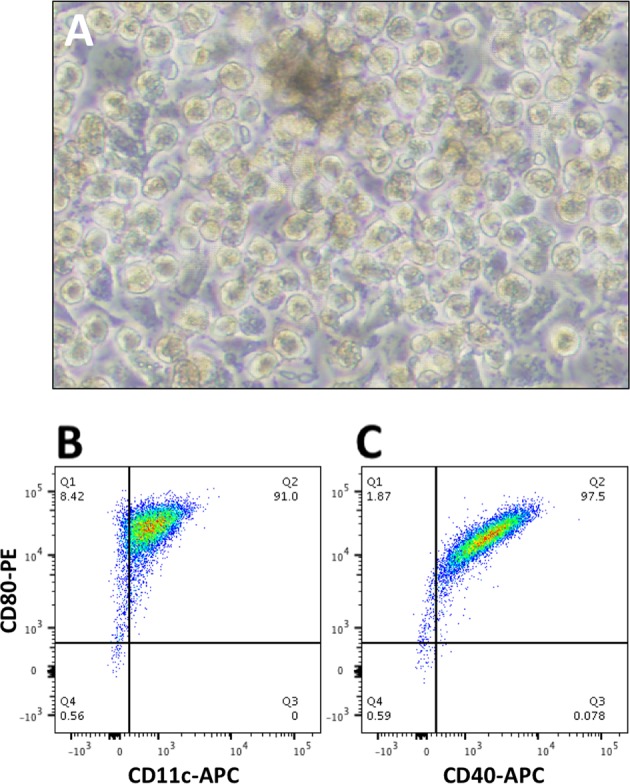
Characterization of canine dendritic cell populations. **a** Mature dendritic cells exhibited a characteristic morphology and expressed high levels of **b** CD80, **c** CD40, and CD11c (both **b** and **c**)

Canine DC were generated from G-CSF-mobilized peripheral blood by plastic adherence of monocytic precursors and 6 days of incubation in GM-CSF and IL-4 as described. Following maturation with canine IL-1β, TNF-α, IL-6, and PGE_2_ [27, 28], the adherent cell population adopted a characteristic dendritic cell morphology (Fig. 1a). Subsequent analysis by flow cytometry indicated that > 90% of the cells in the monocyte gate were CD11c^+^, CD40^+^, and CD80^+^ (Fig. 1b, c), suggestive of a genuine dendritic cell phenotype. Additional flow characterization was not possible at the time due to a lack of commercially-available, canine-reactive antibody reagents.

### Clinical results

Between February 2016 and April 2017, consecutively presenting domestic canines with a histopathologically confirmed diagnosis of hemangiosarcoma and the owners of which could provide written, informed consent were recruited for a phase I open-label pilot study to determine feasibility, safety, and efficacy of a chemo-immunotherapeutic treatment regimen. Eight animals were ultimately enrolled, and five completed the full treatment regimen. Two animals died before treatment could be administered, and one animal was withdrawn from the study by its owners after administration of a single vaccination and without receiving any chemotherapy. The clinical characteristics of each animal are provided in Table 1. Animals are listed in the chronologic order in which they were consented to the study.

**Table 1:** Selected characteristics of enrolled study animals

Subject	Sex	Age (yrs)	Weight (kg)	Breed	Disease stage	Number DC/ dose	DC doses	Total DC/kg	Grade 3 or higher vaccine-related AE	Post-diagnosis OS (Days)	Cause of death
1	Female	13	30.0	Labrador Retriever	III	9.0e6	4	1.2e6	No	480	Acute respiratory distress
2	Male	10	33.0	Labrador Retriever	II	6.6e6	4	0.8e6	No	109	Neoplasia
3	Male	10	12.3	Australian Shepherd	IV	0	0	0	-	35	Neoplasia
4	Female	11	36.4	German Shepherd	IV	0	0	0	-	10	Neoplasia
5	Male	9	28.0	Labrador Retriever	IV	14.0e6	4	2.0e6	No	235	Neoplasia
6	Male	8	13.3	Blue Heeler	IV	8.0e6	1	0.6e6	No	86	Neoplasia
7	Male	10	11.4	Cocker Spaniel	IV	10.0e6	5	4.4e6	No	96	Neoplasia
8	Female	4	28.2	Golden Retriever	II	12.0e6	4	1.7e6	No	105	Neoplasia

**Fig. 2:**
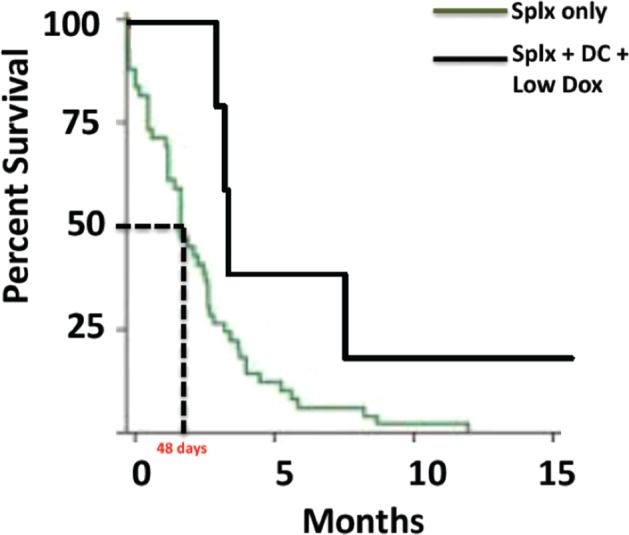
Median survival of animals treated by splenectomy plus chemo-immunotherapy. Differences in survival between treated animals and historical controls were determined by Kaplan–Meier survival analysis. Because study did not contain a parallel control arm, comparison of experimental values with historical controls was achieved by Monte–Carlo simulation assuming a Poisson distribution of hazard rates around a central value of 48 days, itself determined by analysis of 154 historical case controls. Historical control data were adapted from a figure originally published in Wendelburg et al. [11]

Disease burden was evaluated at baseline by ultrasound imaging, echocardiography, and chest X-ray. No animals at baseline exhibited cardiac or lung metastases though one of six animals exhibited local recurrence distal to the site of splenectomy, and three of six exhibited liver metastases at the time of treatment. The other two animals were determined to have no evidence of disease (NED). Of the six animals who began treatment, five received the full treatment regimen of four vaccinations and two cycles of reduced-intensity chemotherapy. One animal was withdrawn from the study by its owners after a single vaccination without undergoing any chemotherapy and was excluded from the per protocol survival analysis. In this analysis (Fig. 2), median survival among the five animals that received the full treatment regimen was 109 days in comparison to a splenectomy-only historical survival of 48 days [11]. Median survival among all eight animals enrolled (intent to treat) was 96 days (not shown). The two animals that were NED at the time treatment was initiated exhibited a mean OS of 107 days (range 105–109 days), whereas the three animals with metastatic or recurrent disease exhibited a mean survival of 270 days (range 96–480 days). Although the study was not statistically powered to determine the significance of these differences, they nonetheless indicate that mean survival was unlikely to be dependent upon disease status at the time of treatment. The lack of a concurrent control arm as well as the significant differences between this study and five comparable studies in the literature prevented determination of reliable conclusions regarding efficacy; however, a statistical comparison of survival in this study to each of five other studies in the literature is provided in Table 2.

**Table 2:** Monte–Carlo estimates of *P* values and 95% confidence intervals in comparison with five historical control studies

Treatment methodology	Comparison group	Study *n*	Half-time (days)	*P* value	Mean ∆ (days)	95% confidence range of ∆	Rosenthal number
	**This study (stages II – IV)**	**5**	**109.0**	**–**	**–**	**–**	**–**
Surgery alone	Wendelburg 2015 stage II & III (harmonic mean)	126	43.3	< 0.00002	65.0	54.3–76.1	> 4.9
	Wood 1998 splenectomy alone *N* = 13 stage I & 19 stage II	32	86.0	0.13	23.9	−18.3–66.1	–
Surgery + chemotherapy	Wendelburg 2015 splenectomy + chemotherapy	54	104.0	0.78	6.7	−34.3–47.7	–
	Moore 2017 splenectomy + anthracycline + lomustine	30	158.0	0.97	−62.6	−254.3–14.7	–
	U’ren 2007 splenectomy + chemotherapy stage II & III	24	133.0	0.34	−24.0	−34.3–47.7	–

**Fig. 3:**
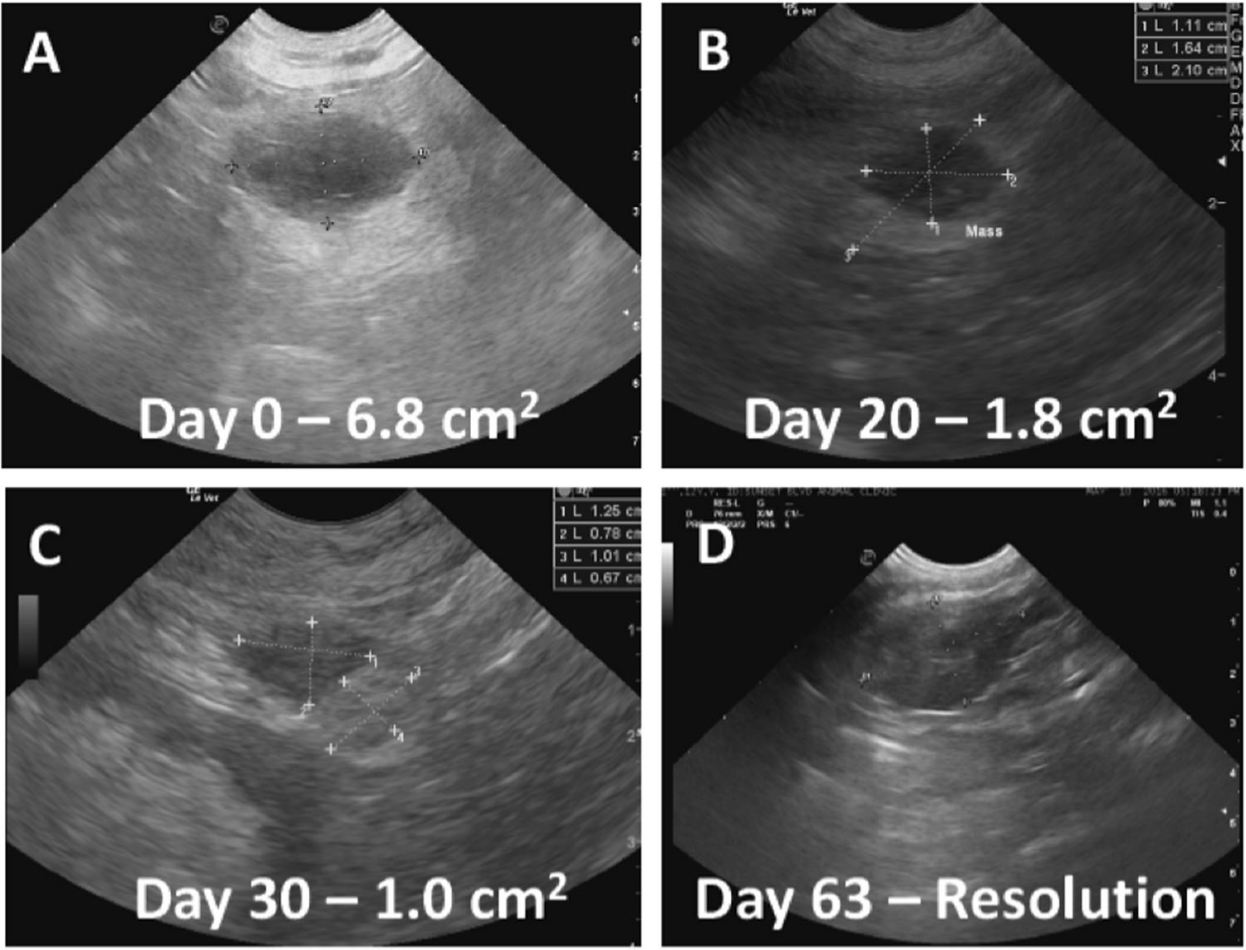
Serial ultrasound imaging documents radiographic evidence of tumor regression. **a** Baseline image at day 0 indicates 6.8 cm mass. **b** Day 20 image indicates mass of 1.8 cm. **c** Day 30 image indicates mass of 1.0 cm with increased evidence of fibrosis. **d** Day 63 image no longer identifiable as a definitive mass

**Fig. 4:**
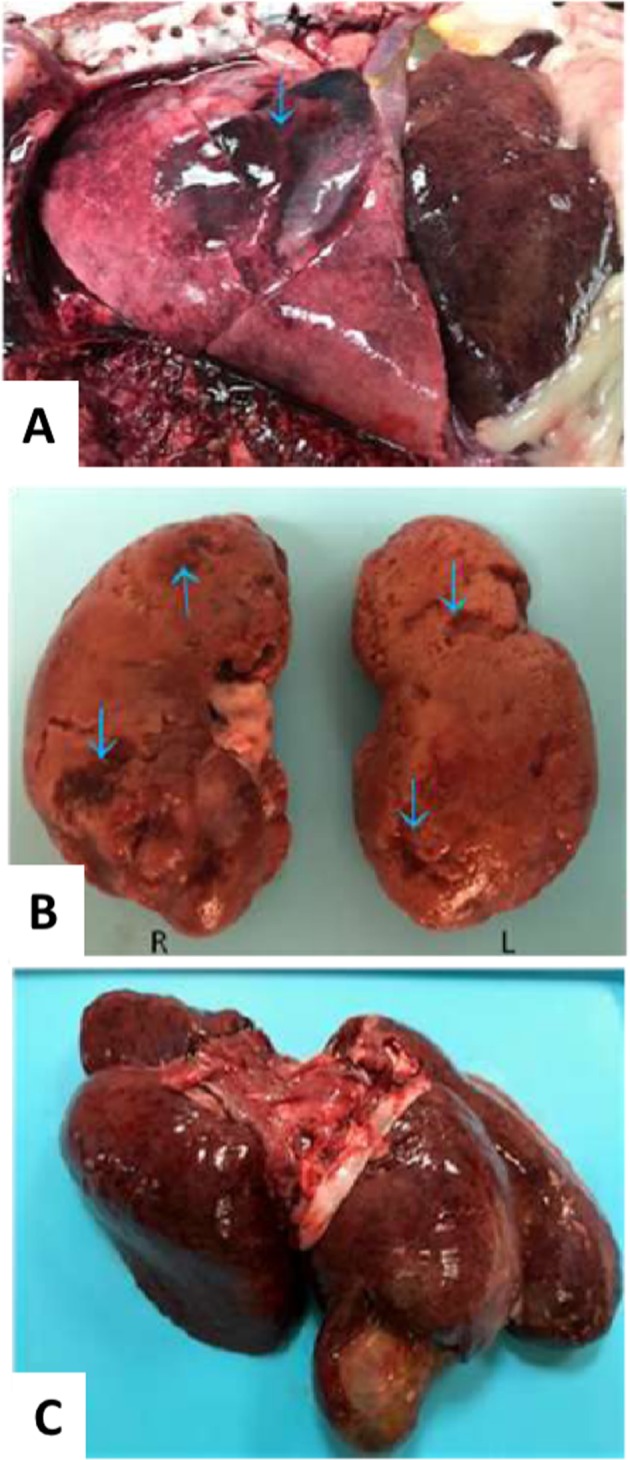
Absence of hemangiosarcoma in select organ systems at necropsy. **a** Right cardiac lobe and part of diaphragmatic lobe of lung. Arrow indicates consolidation. **b** Kidneys: arrows point to age-related cortical pitting and depressions but no hemangiosarcoma was observed. **c** Liver: pale and tan noncancerous subcapsular foci were observed but no evidence of hemangiosarcoma

A single animal was cured of its disease and died tumor-free at 16 months after initiation of treatment. At the time treatment was initiated, this animal exhibited local recurrence near the site of splenectomy. The 6.8 cm recurrent mass was monitored by ultrasound sonography and was observed to resolve radiographically during the first two months of treatment (Fig. 3a–d). By 12 months post treatment, no radiographic evidence of the original mass remained. At 16 months post treatment, the animal was admitted with sudden symptoms of acute respiratory distress and was subsequently euthanized. A full histopathological necropsy was performed to determine cause of death and to rule out recurrent hemangiosarcoma. Examination of the lungs indicated acute, focally extensive pneumonia with pleuritis and numerous cocci and bacilli present. Pneumonia was complicated by moderate to extensive vascular and interstitial amyloidosis [29], which was also present systemically to varying degree in the kidneys, heart, urinary bladder, intestine, and stomach. The animal was also neutropenic and leukopenic. No evidence of recurrent hemangiosarcoma was observed in any organ system (Fig. 4a–c).

### Safety

The animal cured of its disease experienced a single occurrence of grade 1 uveitis ~3 months after treatment was initiated. Uveitis was treated with a 3-week course of ophthalmic corticosteroids and did not recur. This animal was also determined to be suffering from vascular amyloidosis at the time of death from acute respiratory distress and bacterial pneumonia; however, incidence of amyloidosis in the elderly is relatively high and not perfunctorily attributable to other cause [29]. Similarly, neither neutropenia nor leukopenia noted at the time of death were observed by CBC performed during routine follow-up examinations up to 12 months post vaccination. No other adverse events (AE) potentially related to vaccine administration were documented in any of the other enrolled study animals. The precipitating cause of euthanasia in all other study animals was hypovolemic shock and anemia secondary to the presence of actively exsanguinating tumor masses.

## Discussion

HSA is an aggressive, malignant neoplasia with a poor prognosis. Surgery and chemotherapy have exhibited only limited success in prolonging survival times and increasing quality of life in dogs with HSA [30]. Historically, surgery has been the treatment of choice but prolongs survival by a few months at most [31]. As metastasis occurs early in the disease, new treatment modalities that can delay the spread of tumor are indicated. Doxorubicin-based chemotherapy protocols have emerged as the treatment of choice to prevent or contain the metastasis after surgery and to improve the overall survival post splenectomy [32]. Experimental in vitro cell culture [33] and in vivo mouse xenograft [34] evidence also suggest that effects of doxorubicin may be further potentiated by co-administration of reservatrol [35]. Although animals that complete a full course of doxorubicin chemotherapy exhibit a mean OS of 150 days, it has also been proposed that animals surviving long enough to complete the 8-week course of therapy were simply those with slower-progressing tumors as animals that die prior to administration of all five doxorubicin cycles are typically excluded from analysis [11]. In general, the clinical experience with HSA remains highly unsatisfactory, necessitating the need for more efficacious therapeutic approaches. In recent years, immunotherapy has become entrenched as a potent mainstay in the fight against human cancers [25] and is also recognized as a potential treatment option against the canine disease. Vail et al reported the experimental use of liposome-encapsulated immune modulatory agents against canine HSA [36]. De Silva et al. [37] demonstrated that administration of dendritic cell-based immunotherapy in combination with toll-like receptor ligands can resolve systemic and metastatic tumors in dogs. Mito et al. [38] reported efficacy of intra-tumoral dendritic cell-based vaccines in dog tumors when administered in combination with IFN-γ. Gyorffy et al. [39] further demonstrated feasibility and efficacy of an antigen-pulsed dendritic cell-based vaccine against melanoma in dogs.

In this open-label, non-randomized clinical study, we attempted to determine the feasibility of low-dose chemotherapy plus autologous dendritic cell vaccination [22] as a potential therapeutic option for angiosarcoma using canine HSA as a model system. Out of the eight enrolled subjects, five received the complete treatment regimen. Two subjects died before treatment could be administered and one was withdrawn by its owners following the initial vaccination. Among the enrolled subjects that completed the full course of treatment, no serious AE were reported, and median OS was 109 days. This included two long-term survivors demonstrating radiographic evidence of tumor control and/or regression. One of these animals died cancer-free at 16 months subsequent to its initial presentation with HSA. The cause of death in this elderly animal was acute respiratory distress complicated by local and systemic amyloidosis. Pathological amyloidosis is a disease of aging in the developed world, with ~3000 deaths per year attributable to the condition in the United States [29]. Although relatively uncommon among the general population, amyloidosis is estimated to be present in up to 37% of individuals over the age of 80 [40] and has been cited as the cause of death in 70% of the individuals aged 110 or older whom have been autopsied [41]. The 60-pound canine subject in this study was ~14 years old at the time of death, an age physiologically analogous to that of a human between the ages of 70 and 80 [42]. Although we cannot rule out the potential impact of vaccination on the development of amyloidosis, the most likely hypothesis would attribute its manifestation to the natural physiologic consequences of aging.

Our ability to conclude an immunologic hypothesis for the observed clinical results or a survival advantage over current standard of care chemotherapy is very limited. We have previously reported that standard of care chemotherapy, cell-based vaccine immunotherapy, and innate signaling can synergize to elicit enhanced T_H_1 adaptive responses in a mouse model of orthotopic pancreatic ductal adenocarcinoma [22]. Ideally, we would have been able to perform immunomonitoring experiments to validate similar conclusions here; however, the nature of the present study precluded direct immunomonitoring, rendering an immunologic hypothesis speculative. Domestic canines treated in a clinical veterinary setting are not experimental research animals, and we did not treat them as such. Therefore, it is difficult to draw any concrete conclusion beyond affirming feasibility and safety of autologous cell-based immunotherapy in a clinical veterinary setting. Any efficacy comparisons to historical data are speculative given that the abbreviated, low-dose regimen of doxorubicin used here (two cycles of 20 mg/m^2^ here vs. five cycles of 30 mg/m^2^ as standard of care) is not replicated in the historical literature. Further complicating determination of efficacy is that subjects that fail to survive long enough to receive a complete treatment regimen (in one study, the regimen was as long as 150 days) [26] are typically excluded from the analysis, biasing such studies toward inclusion of only long-term survivors. Moreover, although many studies enrolled animals with stage I disease (and its associated advantageous mean OS of 180 days), ours enrolled animals with primarily stage IV disease. Nonetheless, a statistical comparison with each of five appropriate historical studies was provided so that independent determination of efficacy might be gauged in an unbiased manner by the reader.

Despite the obvious shortcomings associated with this small pilot study, anecdotal evidence hinted at potential efficacy and provides a compelling rationale for additional study. Future studies in this area should focus upon enrolling larger numbers of animal subjects as well as performing routine immunomonitoring so as to validate suspected immune-based mechanisms of action. Moreover, the conclusions derived from this investigational new drug (IND)-enabling work imply that human clinical studies may be warranted.

## References

[1] Mendenhall WM, Mendenhall CM, Werning JW, Reith JD, Mendenhall NP (2006). Cutaneous angiosarcoma. Am J Clin Oncol.

[2] Ravi V, Patel S (2013). Vascular sarcomas. Curr Oncol Rep.

[3] Young RJ, Brown NJ, Reed MW, Hughes D, Woll PJ (2010). Angiosarcoma. Lancet Oncol.

[4] Abraham JA (2007). Treatment and outcome of 82 patients with angiosarcoma. Ann Surg Oncol.

[5] Fury MG, Antonescu CR, Van Zee KJ, Brennan MF, Maki RG (2005). A 14-year retrospective review of angiosarcoma: clinical characteristics, prognostic factors, and treatment outcomes with surgery and chemotherapy. Cancer J.

[6] Post SM (2012). Mouse models of sarcomas: critical tools in our understanding of the pathobiology. Clin Sarcoma Res.

[7] Rothweiler S (2015). Generation of a murine hepatic angiosarcoma cell line and reproducible mouse tumor model. Lab Invest.

[8] Alvarez FJ, Hosoya K, Lara-Garcia A, Kisseberth W, Couto G (2013). VAC protocol for treatment of dogs with stage III hemangiosarcoma. J Am Anim Hosp Assoc.

[9] Lana S (2007). Continuous low-dose oral chemotherapy for adjuvant therapy of splenic hemangiosarcoma in dogs. J Vet Intern Med.

[10] Sorenmo K (2007). Clinical and pharmacokinetic characteristics of intracavitary administration of pegylated liposomal encapsulated doxorubicin in dogs with splenic hemangiosarcoma. J Vet Intern Med.

[11] Wendelburg KM (2015). Survival time of dogs with splenic hemangiosarcoma treated by splenectomy with or without adjuvant chemotherapy: 208 cases (2001-2012). J Am Vet Med Assoc.

[12] Wood CA (1998). Prognosis for dogs with stage I or II splenic hemangiosarcoma treated by splenectomy alone: 32 cases (1991-1993). J Am Anim Hosp Assoc.

[13] Dillman R (2004). Phase I/II trial of autologous tumor cell line-derived vaccines for recurrent or metastatic sarcomas. Cancer Biother Radiopharm.

[14] Bol KF, Schreibelt G, Gerritsen WR, de Vries IJ, Figdor CG (2016). Dendritic cell-based immunotherapy: state of the art and beyond. Clin Cancer Res.

[15] Mackall CL (2008). A pilot study of consolidative immunotherapy in patients with high-risk pediatric sarcomas. Clin Cancer Res.

[16] U’Ren LW, Biller BJ, Elmslie RE, Thamm DH, Dow SW (2007). Evaluation of a novel tumor vaccine in dogs with hemangiosarcoma. J Vet Intern Med.

[17] Decker WK (2008). Deficient T(H)-1 responses from TNF-alpha-matured and alpha-CD40-matured dendritic cells. J Immunother.

[18] Decker WK (2009). Th-1 polarization is regulated by dendritic-cell comparison of MHC class I and class II antigens. Blood.

[19] Decker WK (2006). Double loading of dendritic cell MHC class I and MHC class II with an AML antigen repertoire enhances correlates of T-cell immunity in vitro via amplification of T-cell help. Vaccine.

[20] Halpert MM (2016). Dendritic cell-secreted cytotoxic T-lymphocyte-associated protein-4 regulates the T-cell response by downmodulating bystander surface B7. Stem Cells Dev.

[21] V Konduri et al. Genetic adjuvantation of a cell-based therapeutic vaccine for amelioration of chagasic cardiomyopathy. Infect Immun 85, pii: e00127–17 (2017). 10.1128/IAI.00127-17 PMC5563592 28674032

[22] Konduri V (2016). Chemo-immunotherapy mediates durable cure of orthotopic Kras(G12D)/p53(−/−) pancreatic ductal adenocarcinoma. Oncoimmunology.

[23] Liang D, Halpert MM, Konduri V, Decker WK (2015). Stepping out of the cytosol: AIMp1/p43 potentiates the link between innate and adaptive immunity. Int Rev Immunol.

[24] Liang D (2017). AIMp1 potentiates TH1 polarization and is critical for effective antitumor and antiviral immunity. Front Immunol.

[25] Decker WK (2017). Cancer immunotherapy: historical perspective of a clinical revolution and emerging preclinical animal models. Front Immunol.

[26] Moore AS, Rassnick KM, Frimberger AE (2017). Evaluation of clinical and histologic factors associated with survival time in dogs with stage II splenic hemangiosarcoma treated by splenectomy and adjuvant chemotherapy: 30 cases (2011-2014). J Am Vet Med Assoc.

[27] Lee AW (2002). A clinical grade cocktail of cytokines and PGE2 results in uniform maturation of human monocyte-derived dendritic cells: implications for immunotherapy. Vaccine.

[28] Scandella E, Men Y, Gillessen S, Forster R, Groettrup M (2002). Prostaglandin E2 is a key factor for CCR7 surface expression and migration of monocyte-derived dendritic cells. Blood.

[29] Hazenberg BP (2013). Amyloidosis: a clinical overview. Rheum Dis Clin North Am.

[30] Clifford CA, Mackin AJ, Henry CJ (2000). Treatment of canine hemangiosarcoma: 2000 and beyond. J Vet Intern Med.

[31] Brown NO, Patnaik AK, MacEwen EG (1985). Canine hemangiosarcoma: retrospective analysis of 104 cases. J Am Vet Med Assoc.

[32] Finotello R, Stefanello D, Zini E, Marconato L (2017). Comparison of doxorubicin-cyclophosphamide with doxorubicin-dacarbazine for the adjuvant treatment of canine hemangiosarcoma. Vet Comp Oncol.

[33] Rai G, Mishra S, Suman S, Shukla Y (2016). Resveratrol improves the anticancer effects of doxorubicin in vitro and in vivo models: a mechanistic insight. Phytomedicine.

[34] Kim TH (2014). Resveratrol enhances chemosensitivity of doxorubicin in multidrug-resistant human breast cancer cells via increased cellular influx of doxorubicin. Biochim Biophys Acta.

[35] Carlson A., Alderete K. S., Grant M. K. O., Seelig D. M., Sharkey L. C., Zordoky B. N. M. (2017). Anticancer effects of resveratrol in canine hemangiosarcoma cell lines. Veterinary and Comparative Oncology.

[36] Vail DM (1995). Liposome-encapsulated muramyl tripeptide phosphatidylethanolamine adjuvant immunotherapy for splenic hemangiosarcoma in the dog: a randomized multi-institutional clinical trial. Clin Cancer Res.

[37] De Silva NH (2017). Development of effective tumor immunotherapy using a novel dendritic cell-targeting Toll-like receptor ligand. PLoS ONE.

[38] Mito K (2010). IFN{gamma} markedly cooperates with intratumoral dendritic cell vaccine in dog tumor models. Cancer Res.

[39] Gyorffy S (2005). Bone marrow-derived dendritic cell vaccination of dogs with naturally occurring melanoma by using human gp100 antigen. J Vet Intern Med.

[40] Ebert EC, Nagar M (2008). Gastrointestinal manifestations of amyloidosis. Am J Gastroenterol.

[41] Coles LS, Young RD (2012). Supercentenarians and transthyretin amyloidosis: the next frontier of human life extension. Prev Med.

[42] Patronek GJ, Waters DJ, Glickman LT (1997). Comparative longevity of pet dogs and humans: implications for gerontology research. J Gerontol A Biol Sci Med Sci.

